# A parasite’s take on the evolutionary cell biology of MICOS

**DOI:** 10.1371/journal.ppat.1008166

**Published:** 2019-12-19

**Authors:** Hassan Hashimi

**Affiliations:** Institute of Parasitology, Biology Center, Czech Academy of Sciences and Faculty of Science, University of South Bohemia, České Budějovice, Czech Republic; University at Buffalo School of Medicine and Biomedical Sciences, UNITED STATES

*Trypanosoma brucei* has been a subject of ardent scrutiny since it was shown at the turn of the 20th century to be the causative agent of human African trypanosomiasis, then known as sleeping sickness, and Nagana in cattle. Technological advances that began around the start of the 21st century allowed *T*. *brucei* to emerge as a bona fide model organism, with an effective and versatile genetic toolkit that facilitates molecular dissection of these fascinating parasites [1]. Other technological advances emerging at the same time allowed biologists to construct a consensual eukaryotic tree of life [2]. This tree (Fig 1) supported *T*. *brucei* and related euglenozoans forming their own major clade (Discoba), only distantly related to supergroups like Opisthokonta, which encompasses fungi and animals, and Archaeplastida, which contains land plants. This means that, far from being primitive eukaryotes, trypanosomes have evolved independently of any other model organism for over an eon. Thus, *T*. *brucei* and its ilk are not just intrinsically interesting parasites but also attractive models for investigating the evolution of fundamental molecular and cellular biology of eukaryotes from a detached perspective. We have recently delved into the evolutionary cell biology (ECB) of the ancient mitochondrial contact site and cristae organization system (MICOS) protein complex in *T*. *brucei* to better understand how it contributes to the architecture of an ancient organelle, the mitochondrion [3,4].

**Fig 1:**
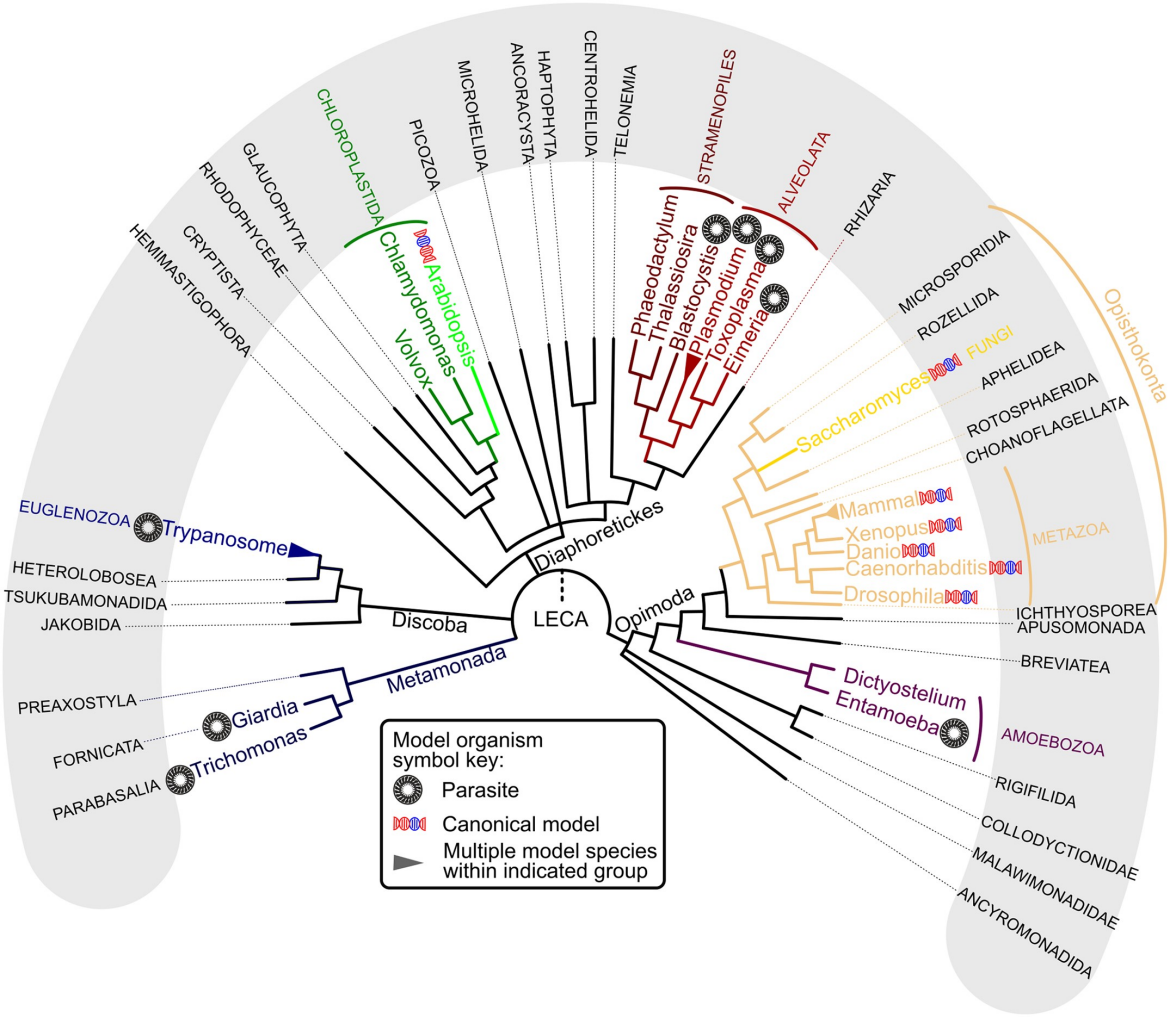
Distribution of model organisms in the current eukaryotic tree of life. Model systems capable of transgenic manipulation (according to evidence in the literature) that are considered to be typical canonical models and parasites (designated as in symbol key below tree) are indicated at ends of branches, usually by genus name. The exceptions are “Trypanosome” and “Mammal,” which contain several genera that are indicated in Fig 3. Names of lineages demarked by branches are given in outside grey semicircle. Dashed branch to LECA at root of tree indicates its ambiguous position in the current tree. Only supergroups that are discussed here are highlighted. Larger formal groups are labeled at the root of their respective subtrees. LECA, last eukaryotic common ancestor.

## What is evolutionary cell biology?

Half of the 2018 Nobel Prize in Chemistry was awarded to Frances Arnold for the directed evolution of proteins [5]. This methodology, inspired by natural selection, harnesses the exploration of random mutations in the laboratory to tailor existing enzymatic activities to produce beneficial compounds for biomedicine and biotechnology. This recognition of directed evolution’s promise to create hitherto only imagined chemicals for specific tasks at human timescales is certainly well deserved. Yet the evolutionary forces shaping the bewildering organismal diversity that has radiated from the last eukaryotic common ancestor (LECA) on an approximately 1.9 giga-annum (Ga) geological timescale [6] remains underappreciated (Fig 1).

The emerging field of ECB embraces eukaryotic diversity in order to understand the fundamental biology of eukaryotes [7]. ECB postulates that there are specific physical and chemical restraints to biological systems that confine natural selection [7]. This in turn explains the persistence of particular cellular processes and structures, plus their key molecular components, throughout the eukaryotic tree. Alternatively, analogous traits can independently emerge in isolated lineages in order to overcome universal physicochemical constraints by convergent evolution [8]. ECB also allows the identification of more flexible parts of a biological system free of any restrictions, from which novel innovations can emerge via divergent evolution.

Indeed, the view purveyed by ECB has given biologists insight into the general workings of ancient eukaryotic structures, such as the kinetochores needed for chromosome segregation [9], the nuclear pore complex at the interface of cytosolic expression of nuclear DNA [10], the endomembrane system that facilitates secretion of some of those proteins [11], and macromolecular complexes that import another subset of proteins into the mitochondrion [12,13,14]. Furthermore, the conservation of complex processes and structures among the disparate cells that constitute eukaryotes implies that LECA was already a complex cell, already possessing the aforementioned elements among others to facilitate a sophisticated lifestyle [15]. Thus, as the singularity of eukaryotic diversity, LECA likely encapsulates the fundamental biology of extant eukaryotes, something ECB aims to reveal.

## What is MICOS?

Mitochondria are among the organelles inferred to be possessed by LECA, arising from an alphaproteobacterial endosymbiont at some point during eukaryogenesis [16]. Famously, mitochondria are known as the powerhouse of the cell (to the point that this phrase has been co-opted as an internet meme) as they are the site of oxidative phosphorylation, the combustion of carbon sources to generate ATP in aerobic conditions. While this function has been lost in certain anaerobic lineages, there is ample evidence that LECA’s mitochondrion had this capability [16].

**Fig 2:**
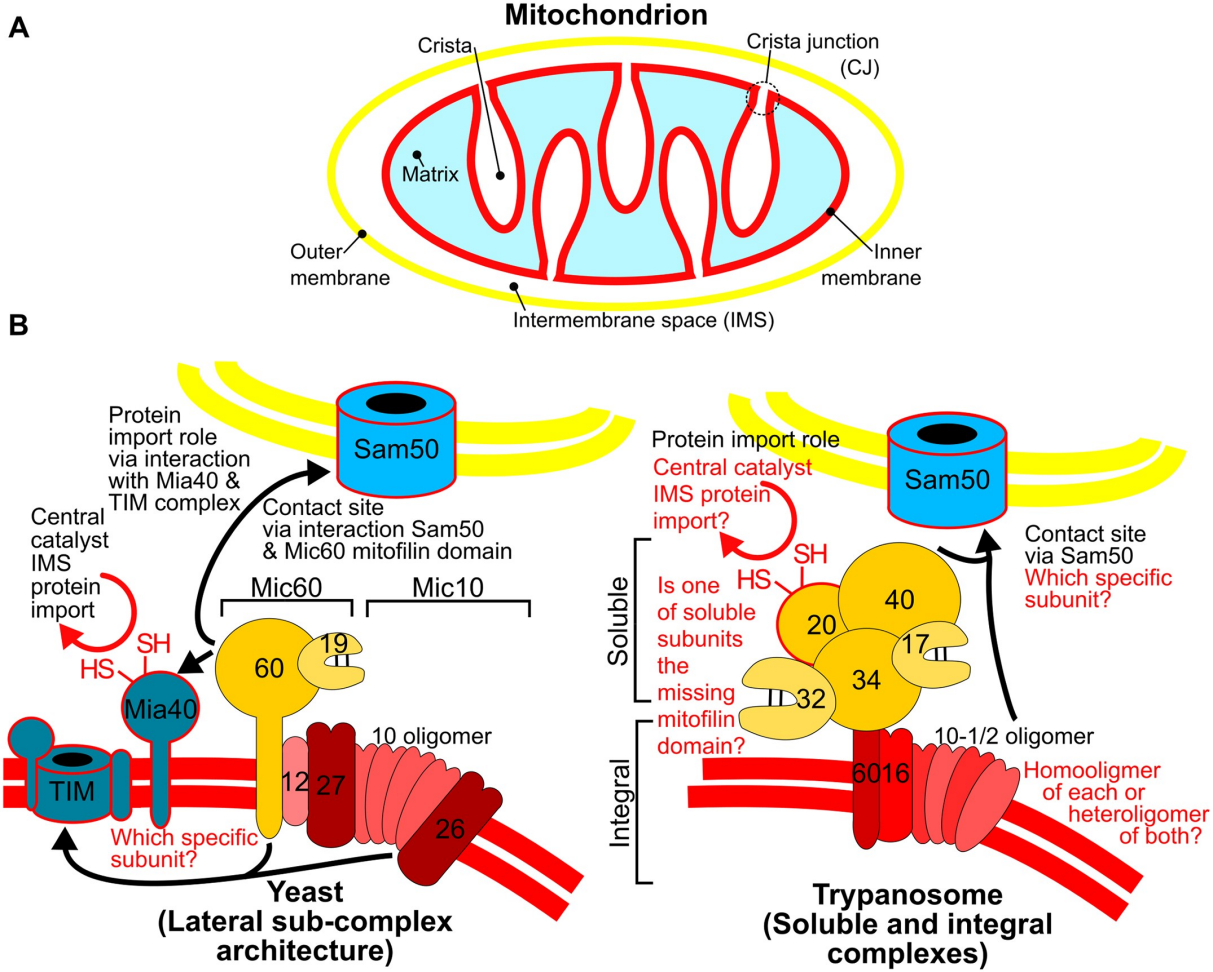
Conserved and diverged features of yeast and trypanosome MICOS. (A) Basic mitochondrial architecture with relevant subcompartments indicated. (B) Comparison of MICOS complexes from yeast and trypanosomes. Color of outer and inner membrane phospholipid bilayers (double lines) as in (A). Subcomplex architecture indicated by brackets and color of component subunits. Only the numbers used in naming the MICOS subunits by the MicXX convention [22] are given. Conserved features of 2 complexes in black text with remaining uncertainties in red text. Extra-MICOS interactions indicated by back arrows pointing to interacting entity. Proteins involved in mitochondrial protein import in blue with red outline. CJ, crista junction; HS/SH, cysteine thiol side chain; IMS, intermembrane space; MICOS, mitochondrial contact site and cristae organization system; TIM, translocase of the inner membrane.

The protein machinery responsible for oxidative phosphorylation resides in cristae, invaginations of the inner of the 2 mitochondrial membranes (Fig 2A) [17]. Cristae are connected to the inner membrane by small stems at their base called crista junctions (CJs) [18]. CJs are also where the inner and outer membranes come into close apposition to form a contact site (CS) [19], although some CSs have been observed away from CJs [18].

The discovery of the multiprotein complex responsible for CJ formation and maintenance in budding yeast was reported in 2011 [19,20,21], later dubbed MICOS [22]. Deletion of key MICOS subunits results in the loss of CJs and consequent detachment of elongated cristae into the innermost mitochondrial compartment, the matrix. Consistent with this effect on cristae, MICOS deletion mutants were unable to grow in conditions that force them to rely on oxidative phosphorylation. This phenotype was ameliorated when they were grown on fermentable sources of energy. To facilitate interaction with the mitochondrial outer membrane, MICOS interacts with the outer membrane protein Sam50 (also known as Tob55) and the translocase of the outer membrane (TOM), both of which play central roles in mitochondrial protein import [19,20]. This outer membrane protein interaction provides a molecular basis for MICOS-mediated CS formation. In addition, MICOS has been shown to interact with Mia40 and the translocase of the inner membrane (TIM) 23 complex, which facilitate import into the intermembrane space (IMS) and matrix, respectively [21,23]. Thus, MICOS plays a multifarious role in shaping mitochondria.

## What does trypanosome MICOS tell us?

A thorough survey of genomes found across the eukaryotic tree revealed that the presence of genes encoding the core MICOS subunits Mic10 and Mic60 coincides with the occurrence of cristae-bearing aerobic mitochondria [24], implying that these 2 proteins were already shaping cristae in LECA. More surprising still was that Mic60 was even found in certain extant alphaproteobacterial lineages, suggesting its origin from the endosymbiotic ancestor of mitochondria. However, the other 4 MICOS subunits identified in yeast were not always found outside of opisthokonts.

Because the cristae shaping role of MICOS outside of opisthokont model organisms was still hypothetical and the bioinformatics search for novel MICOS subunits was saturated, we undertook the task of characterizing MICOS in *T*. *brucei* in the name of ECB [3]. Another interesting aspect of this comparison is that yeast and most other opisthokonts have plate-like lamellar cristae, whereas trypanosomes typically bear paddle-like discoidal cristae [24]. Perhaps some potential novelties of trypanosome MICOS may contribute to the discoidal shape?

When looking for an appropriate affinity handle to capture the *T*. *brucei* complex, we encountered the first of many surprises about trypanosome MICOS: A gene encoding the highly conserved Mic60 appeared to be missing, whereas 2 Mic10 paralogs were found in all trypanosomatids [3,24]. Simultaneous ablation of the 2 Mic10 paralogs resulted in CJ loss and the accumulation of elongated and stacked cristae in the matrix, confirming the conservation of Mic10’s role in CJ formation and maintenance. It also reassured us that we were on the right path to isolating the first MICOS complex outside of Opisthokonta.

The 2 Mic10 paralogs interacted with the same 7 proteins using 2 different immunoprecipitation strategies [3]. We went on to show that some of the other newly discovered subunits, namely Mic60, Mic20, and Mic34, also co-immunoprecipitated the same cohort of proteins. Final confirmation that we obtained trypanosome MICOS came by demonstrating that all 9 subunits were assembled into a >1 megadalton (MDa) complex and enriched within crista membranes.

This information allows us to compare the MICOS complexes from *T*. *brucei* and yeast (Fig 2B), species that have evolved in independent lineages for approximately 1.5 Ga [6]. Over this timescale, MICOS maintained its fundamental roles in formation of both CJs as well as CSs, the latter supported by *T*. *brucei* MICOS interaction with outer membrane Sam50. The functional significance of MICOS-mediated CSs in yeast is to facilitate both mitochondrial protein import as well as lipid biosynthesis and trafficking [17,21,25]. Whether this is true for trypanosomes remains an open question. Furthermore, at least one paralog of the core Mic10 subunit appears to form oligomers as in yeast [26,27], although it remains unanswered whether these are homooligomers or assemblies of both paralogs [4]. The observation that the levels of the Mic10-1 paralog is reduced upon Mic10-2 deletion does suggest that they indeed come together to form heteroligomers [4]. Of course, this begs the question of why there are 2 paralogs in trypanosomes while yeast and most other eukaryotes have a single Mic10 [3,24]. Further work will hopefully answer questions, such as whether does the presence of these 2 Mic10 paralogs contributes somehow to the shape of discoid cristae. Also, does their apparent interdependence represent some kind of adaptation in trypanosomes, or is it simply a fluke that eventually became entrenched by neutral evolution [28]?

Several differences between yeast and trypanosome MICOS may also prove to be useful to begin to understand the evolutionary history of the complex (Fig 2B). Unlike the yeast complex, which has a single soluble subunit within the IMS called Mic19, the bulk of the *T*. *brucei* MICOS subunits are soluble IMS proteins [3]. Two of them even have twin disulfide bonds that are a feature of Mic19 and other IMS proteins, although it remains unclear whether one or both are true homologs or analogs of Mic19. The soluble subunits of *T*. *brucei* MICOS are organized into a separate subcomplex from the one formed by the 4 integral membrane subunits [4]. This is in contrast to the architecture of yeast MICOS, which is divided into 2 membrane-embedded subcomplexes, each organized around either Mic10 or Mic60, the 2 core subunits [17]. The subcomplex organization of *T*. *brucei* MICOS may be a consequence of the lack of a Mic60 bearing the characteristic mitofilin domain [24]. One of the integral subunits retains conserved structural elements present in all Mic60 orthologs despite not having a mitofilin domain, prompting us to provisionally call it Mic60 [3]. We speculate that one of the soluble subunits interacting with this integral subunit may represent the missing mitofilin domain.

Among the novel soluble subunits found in *T*. *brucei* MICOS is a thioredoxin-like protein we call Mic20 [3]. We were astonished to find that Mic20 down-regulation resulted not only in aberrant cristae but also a depletion of IMS proteins. We hypothesize that we may have serendipitously found among the trypanosome MICOS subunits the hitherto missing analog of Mia40, the central catalyst responsible for import of proteins into IMS by oxidative folding [29,30]. Ablation of the Mic20-containing soluble subcomplex results in defective protein import into the matrix [4]. This may be a downstream effect since *T*. *brucei* TIM complex assembly requires small IMS proteins that are maturated by oxidative folding [13,14]. Our unexpected observation that subunits of MICOS belonging to the soluble subcomplex are needed for normal growth in media in which oxidative phosphorylation is superfluous is consistent with the complex playing an essential role in mitochondrial protein import [3,4], an essential process [12]. While the presence of a stably interacting subunit involved in mitochondrial protein import is ostensibly a novel feature of trypanosome MICOS, there is evidence in yeast that MICOS interacts with machinery involved in import into the IMS and matrix [21,23]. Thus, it appears that MICOS involvement in mitochondrial protein import may too represent a fundamental role for this ancient complex, even if the degree to which the complex’s integration into this process may differ among eukaryotes.

## How can parasites serve the cause of evolutionary cell biology?

We have used *T*. *brucei*, a parasite transformed into a laboratory model organism, to expand our perspective on MICOS outside of knowledge gained from canonical opisthokont models. This has allowed us to identify conserved and diverged features of MICOS orthologs that have undergone independent evolution for over an eon. This and other studies on conserved cellular structures, such as those previously mentioned [9,10,11], demonstrate the value of combining data acquired from noncanonical model organisms with those from conventional ones to reveal the fundamental biology of eukaryotes.

**Fig 3:**
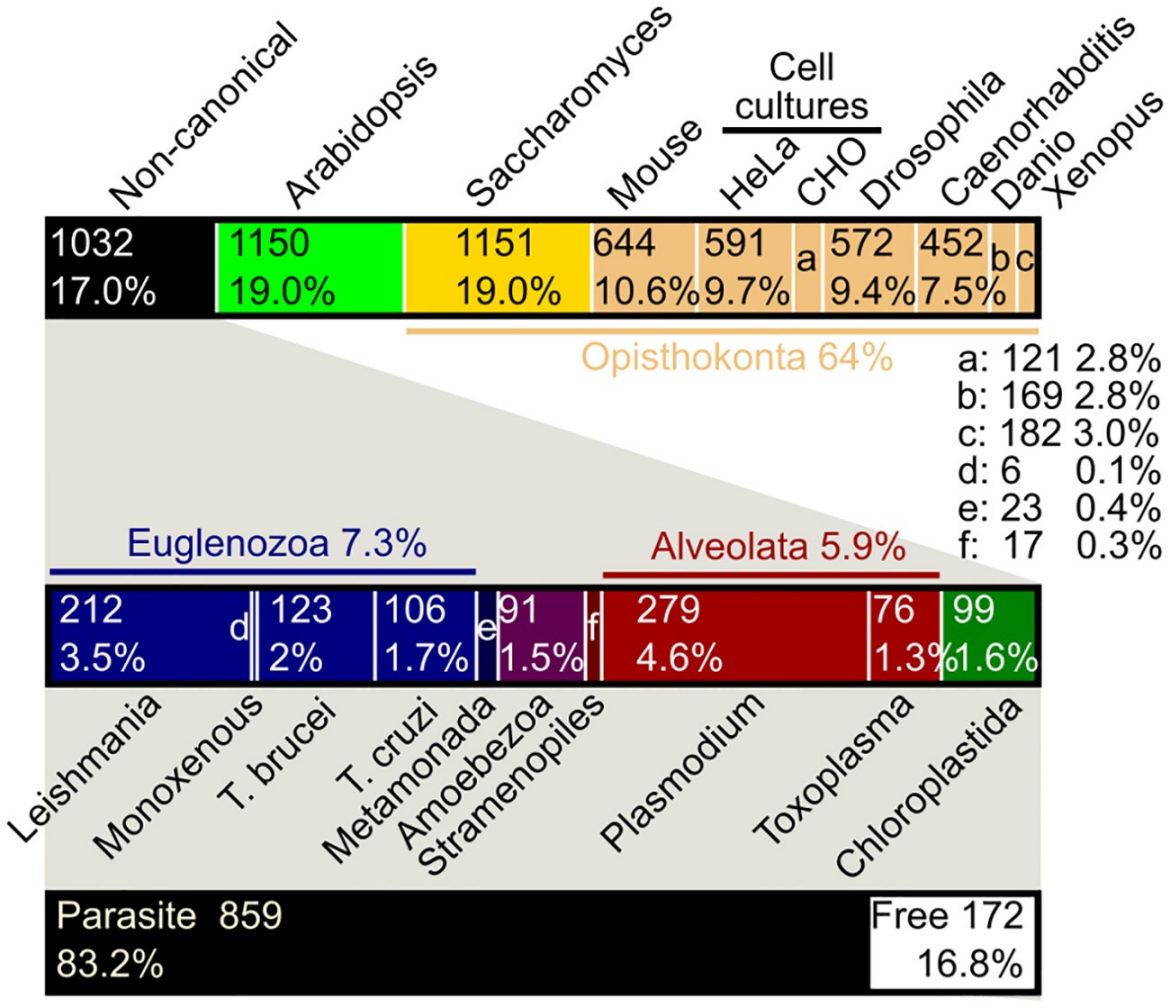
Distribution of “Molecular Biology and Biochemistry” and “Cell Biology” 2018 primary research articles from conventional and noncanonical model organisms. The Web of Science database was searched for each model system mentioned here and in Fig 1 in the year 2018, excluding review articles. The number of articles in these 2 categories was recorded, with articles that were categorized as both “Molecular Biology and Biochemistry” and “Cell Biology” collapsed into one entry. Top bar is the distribution of all articles, while middle bar is a detail showing these statistics for noncanonical model organisms. The bottom bar depicts how many articles come from noncanonical models that are parasites (in black) and free-living organisms (“Free,” white), with percentage (of total noncanonical models) and total article numbers as in the other 2 bars. Total numbers of articles and percentages of total articles from all model organisms are given on top and bottom, respectively, of each category box. Boxes that are too small to report these statistics are demarked with a small letter; their statistics are given between top and middle bars. Monoexenous refers to cultured insect trypanosome species. CHO, Chinese hamster ovary cell line.

A cursory look into Web of Science for research articles published in 2018 dealing with the biochemistry plus molecular and cell biology of conventional and noncanonical models shows that our knowledge about these topics is heavily skewed toward just 2 lineages (Fig 3). Papers reporting results from model organisms belonging to Opisthokonta composed 64% of molecular and cell biology articles while those from *Arabidopsis thaliana* represented 19%. Only 17% of papers dealing with these topics came from data generated with noncanonical systems. Thus, our knowledge about the fundamental molecular and cell biology of eukaryotes is heavily biased toward data from a handful of models mainly belonging to Opisthokonta.

About 80% of articles from noncanonical systems are from parasites, mostly trypanosomes and apicomplexans. This agrees with the overlap of noncanonical models being developed from parasitic organisms, with only a smattering of free-living exceptions (Fig 1). The current paucity of free-living noncanonical models has forced molecular parasitology into the forefront of ECB by default. A major focus of molecular parasitology has been to identify biological deviations between the host and pathogen to exploit in the treatment of infectious diseases. However, molecular parasitologists should also keep in mind that they are by virtue also uncovering biological data that are relevant for the ECB cause. Such contributions lead to a more balanced knowledge base of the fundamental molecular and cellular processes underlying eukaryotic biology. Not only would this help us to understand LECA, and thus the basic architecture of eukaryotes, but may also help us to better understand certain processes, which in turn may lead to treatments of diseases that are not caused by these parasites.
